# How social capital enabled healthcare access and navigation by vulnerable people during Covid-19

**DOI:** 10.1093/eurpub/ckac131.113

**Published:** 2022-10-25

**Authors:** F Zanni

**Affiliations:** 1 Public Health, University of Bologna, Bologna, Italy; 2 Global Public Health, Karolinska Institutet, Stockholm, Sweden

## Abstract

**Background:**

Healthcare systems have become complex and fragmented, negatively affecting healthcare access and navigation. This is especially the case for socio-economically vulnerable people, who encounter organisational and administrative hindrances trying to access care. These difficulties have worsened during Covid-19. Scholarly literature recognises that - by moulding navigation practices - social capital may mediate between potential and realised access to healthcare. However, little is known about how this mediating work practically unfolds.

**Methods:**

This case study aimed to understand how social capital might affect healthcare navigation practices. To do so, we investigated how the People’s Health Lab (PHL), a community-based organisation, supported socio-economically vulnerable people in navigating healthcare during the Covid-19 pandemic in Bologna, Italy. Nine months of participant observation were conducted both in person and digitally from July 2020 to March 2021. Twelve semistructured interviews were also conducted with volunteers of the organisation. Fieldnotes and interview transcripts were analysed through Thematic Analysis.

**Results:**

PHL support activities addressed barriers to healthcare navigation by vulnerable people, which were found to be services fragmentation, bureaucracy and Covid-19 restrictions. Volunteers of the PHL connected vulnerable individuals to health services in manifold, flexible ways, working without standard operative protocols and relying on informal personal contacts within public services. This was found to be key in enabling navigation of healthcare during the first three waves of the pandemic.

**Conclusions:**

Our study provides evidence about how structural, linking social capital - the material and nonmaterial resources mobilised through the relationships between heterogeneous groups (the People’s Health Lab, health authorities and vulnerable people) - can mediate access to fragmented healthcare systems by shaping navigation practices.

**Key messages:**

• Contemporary healthcare systems – including universal ones – have become complex and fragmented, posing access and navigation challenges to their users, especially those socio-economically vulnerable.

• Linking social capital can mediate access to fragmented healthcare systems by flexibly mobilising material and nonmaterial resources through informal relationships between heterogeneous groups.

